# Salivary Proteomics Reveals Oxidative Markers in E‑Cigarette Users

**DOI:** 10.1021/acs.jproteome.5c00347

**Published:** 2026-03-09

**Authors:** Natalia de Carvalho Faria, Bruna Fernandes do Carmo Carvalho, Monica Ghislaine Oliveira Alves, Ismael Feitosa Lima, Leo Kei Iwai, Murilo Salardani, André Zelanis, Janete Dias Almeida

**Affiliations:** † Department of Bioscience and Oral Diagnosis, Institute of Science and Technology, São José dos Campos, São Paulo State University (UNESP), São José dos Campos, São Paulo 12245-000, Brazil; ‡ Laboratory of Applied Toxinology (LETA), Center of Toxins, Immune-Response and Cell Signaling (CeTICS), 534834 Butantan Institute, São Paulo, São Paulo 05503-900, Brazil; § Functional Proteomics Laboratory, Institute of Science and Technology, 28105 Federal University of São Paulo (ICT-UNIFESP), São José dos Campos, São Paulo 12231-280, Brazil

**Keywords:** proteome, saliva, electronic cigarette users, electronic nicotine delivery systems, glutathione transferase

## Abstract

Electronic cigarettes (e-cigs) have become increasingly popular, particularly among younger populations. This study aimed to evaluate the salivary proteome of e-cig users and identify potential alterations in saliva composition. Participants were divided into the Electronic Cigarette Group (EG, *n* = 25 regular exclusive users) and the Control Group (CG, *n* = 25 nonsmokers/nonusers), matched by sex and age. Clinical examination and unstimulated saliva collection were performed for proteomic analysis. A total of 1,773 proteins were identified, of which 92 had consistent quantitative values between groups. Comparison using the Wilcoxon–Mann–Whitney test revealed 22 proteins with differential abundance (*p* ≤ 0.05), most of them up-regulated in EG, notably Peroxiredoxin-1, while few showed decreased abundance. Functional enrichment analysis highlighted pathways related to keratinization, keratinocyte differentiation, and stress response, suggesting activation of cellular defense and adaptation mechanisms. These results indicate that e-cig aerosol exposure induces biological alterations in the oral environment, including modulation of proteins linked to oxidative stress and epithelial integrity. Overall, the findings emphasize the need for long-term monitoring of e-cig users and reinforce the importance of educational strategies to counter the perception of low risk associated with electronic cigarettes.

## Graphical abstract

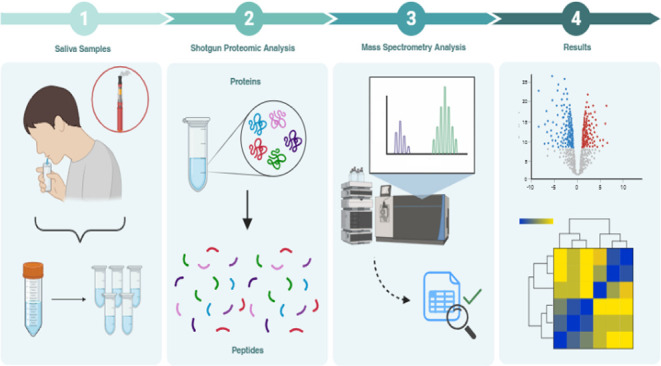

## Introduction

The use of Electronic Nicotine Delivery Systems (ENDS), popularly known as electronic cigarettes (e-cigs) or vapes, reached an impactful global public health problem. These devices have emerged as an alternative to conventional cigarettes, and their consumption has increased exponentially over the years especially among young people.^1^ This popularity and high social acceptability of e-cigs are attributed to the use of highly palatable and pleasant-smelling flavorings, and to their marketing, which presents them as modern and safe, as well as the use of highly palatable flavorings.^1^

The use of e-cigarettes as support to smoking cessation is a very controversial subject. However, scientific evidence has already shown that, in addition to not being efficient in this process and increasing nicotine dependence, they are also not as harmless to health as previously thought.^2,3^

Their flavorings or e-liquids typically consist of a mixture of water, flavorings, nicotine in varying concentrations, propylene glycol, and vegetable glycerin.^4^ These products may also contain substances considered cytotoxic and/or carcinogenic, including nitrosamines, heavy metals, formaldehyde, acetaldehyde, acrolein, polycyclic aromatic hydrocarbons and acetones.^5,6^ Inhalation of these compounds has harmful effects on the respiratory and cardiovascular systems, as well as on the oral mucosa and saliva.^7–9^

Saliva is a biological fluid composed of water, proteins, inorganic substances, and organic substances.^10^ Reduced salivary function can have various oral health consequences, including difficulty swallowing and speaking, an increased risk of dental caries, oral mucosal lesions, opportunistic fungal infections, and periodontal disease.^11–13^

In addition to being a complex and multifunctional biofluid, saliva is easily collected, noninvasive, low cost, and relatively easy to transport and store.^14^ Salivary samples have allowed the identification of most diverse diseases, including oral squamous cell carcinoma. In this sense, also have been described promising biomarkers regarding the harmful, inflammatory and oxidative stress of smoke tobacco and hookah.^15–17^

It contains more than 2000 proteins and peptides involved in various biological functions in the oral cavity.^18^ Identifying and quantifying these proteins can improve the ability to provide more specific diagnoses and prognoses, assist in selecting the best individualized treatment, and monitor patient responses.^19^ Therefore, the objective of the present study was to analyze the differences in the saliva proteome profile between users of electronic cigarettes and a control group. Additionally, the study aimed to investigate possible salivary alterations in individuals using electronic cigarettes.

## Results

### Participant Demographics and Baseline Characteristics

This work draws on the same participant cohort examined in our previous metabolomic study of e-cigarette users.^16^ Participant characteristics, including demographic variables and vaping-related behaviors, have been described elsewhere and are summarized in Supplementary Tables S1 and S2. These data are reused in accordance with the Creative Commons Attribution (CC BY 4.0) license of MDPI. Additional details on sociodemographic and consumption patterns are provided in the Supporting Information.

The study population was mainly composed of men (56%), with mean ages of approximately 26–27 years. Both groups showed comparable epidemiological profiles, characterized by a predominance of self-reported white participants and a high educational level (Table S1).

Compared with the control group, the e-cigarette group exhibited higher levels of exhaled carbon monoxide (2.12 ± 1.59; *p* = 0.015) and reduced peripheral oxygen saturation (96.76 ± 1.23; *p* = 0.036), as shown in Table S1.

Regarding the profile of alcoholic beverage consumption, the EG showed higher results. Although the overall mean AUDIT (Alcohol Use Disorders Identification Test) score for both groups remains in the “low-risk” category, the EG mean was almost double that observed in the CG (*p*-value = 0.003) (Table S1). When comparing the mean number of alcohol doses consumed, a clear pattern of higher consumption in the EG was observed, where 40% of the CG participants consumed between 1 and 2 doses, while 40% of the EG participants consumed a minimum of 3 to 4 doses (Table S1).

### Salivary Analyses

Salivary measurements showed that the e-cigarette group presented lower viscosity values (2.04 ± 1.33; *p* = 0.048) and markedly higher cotinine levels than the control group. No statistically significant differences were observed for the remaining salivary parameters analyzed (Table S1).

### Electronic Cigarette Use

The usage pattern in the EG is detailed in Table S2. Participants in the EG had been using the device for approximately 2.13 years. In this group, usage was daily for 52% of the individuals, with 60% using the device from 7 up to more than 10 times per day. Fruity/sweet flavorings were the most chosen, followed by menthol. Regarding dosage, the mean nicotine concentration observed was 37.2 mg/mL, a value considered very high.

A relevant finding refers to the association between e-cig and alcohol. Approximately 76% of participants reported the concomitant use of alcoholic beverages and electronic cigarettes. Furthermore, 52% stated that alcohol consumption was linked to an increase in the frequency of device use (Table S2).

### Proteomic Analysis

A total of 1,773 proteins were identified, as detailed in Supplementary Tables S3 and S4. As expected for data-dependent acquisition proteomics, a high proportion of missing values was observed across samples, reflecting stochastic precursor selection and limited detectability of low-abundance proteins. Given the dominance and variability of missingness in our data set, imputation would have a disproportionate impact on downstream statistical analyses and could artificially drive group differences. For this reason, we opted for a more stringent filtering strategy that prioritizes robustness and interpretability over proteome coverage. While this reduces the total number of retained proteins, it minimizes the risk of imputation-driven artifacts and ensures that the reported results are based on consistently observed quantitative measurements. Therefore, after data processing and filtering (i.e., considering proteins that were identified in all subjects of each experimental group), 92 proteins showed quantitative values in both EG and CG, as detailed in Supplementary Table S5 and Supplementary Figure S2. A heatmap illustrating the abundance profiles of all 92 quantified proteins is shown in Figure 1 and detailed in Supplementary Table S5.

**Figure 1:**
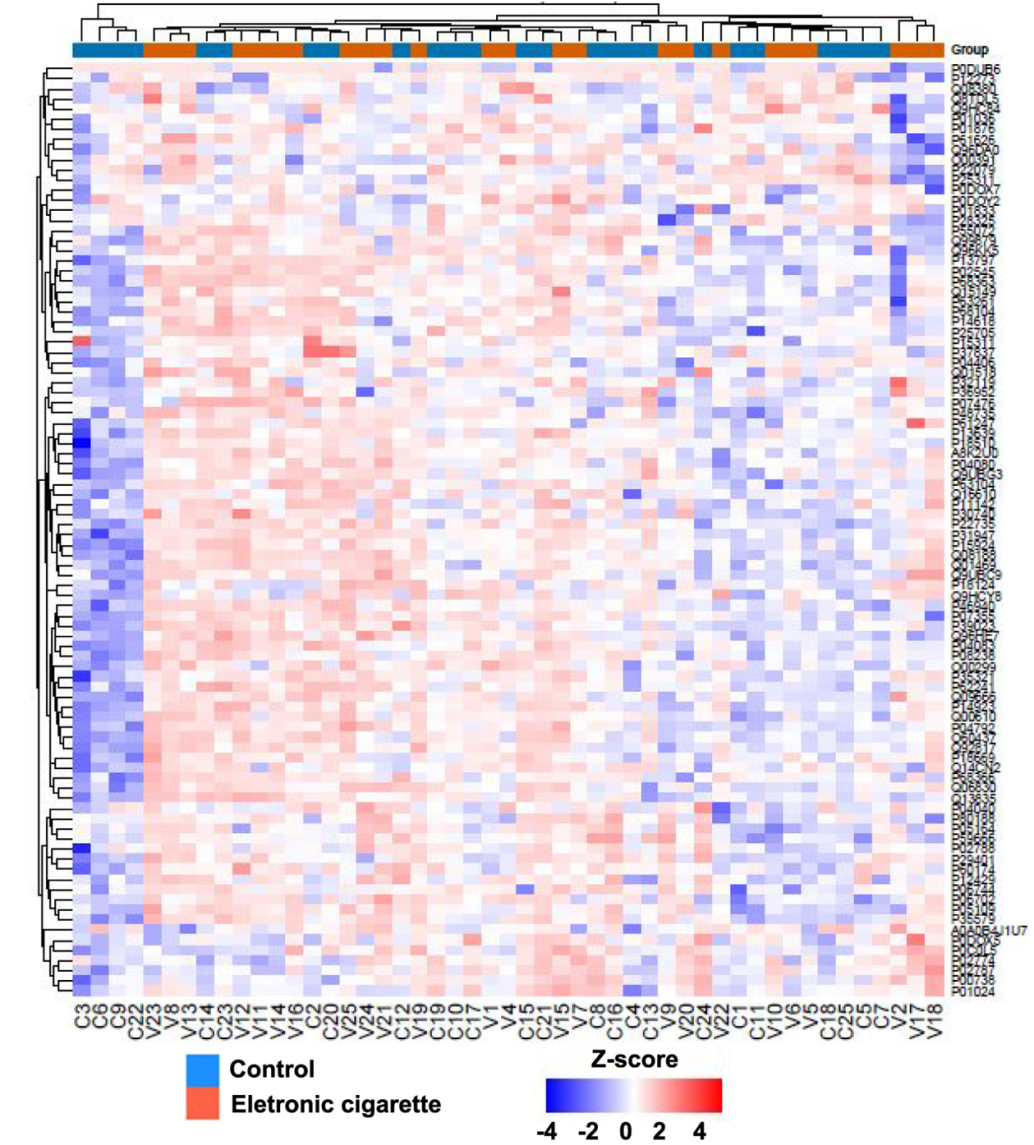
Groups and proteins evaluation. Heatmap of the log_2_-transformed intensity values for the 92 proteins shared by all subject from EG (red) and CG (blue).

Differences between groups were assessed using the Wilcoxon–Mann–Whitney test, applied independently to each protein. In total, 22 proteins were found to be statistically significant (*p* ≤ 0.05). As visualized in the network representation (Figure 2A), there was a predominance of proteins that showed an increase in their abundance (*up-regulation*) in the EG saliva compared to the CG, with the Peroxiredoxin-1 (Q06830) protein being highlighted. In contrast, a smaller number of proteins presented a decrease in their abundance (*down-regulation*) in the EG.

**Figure 2:**
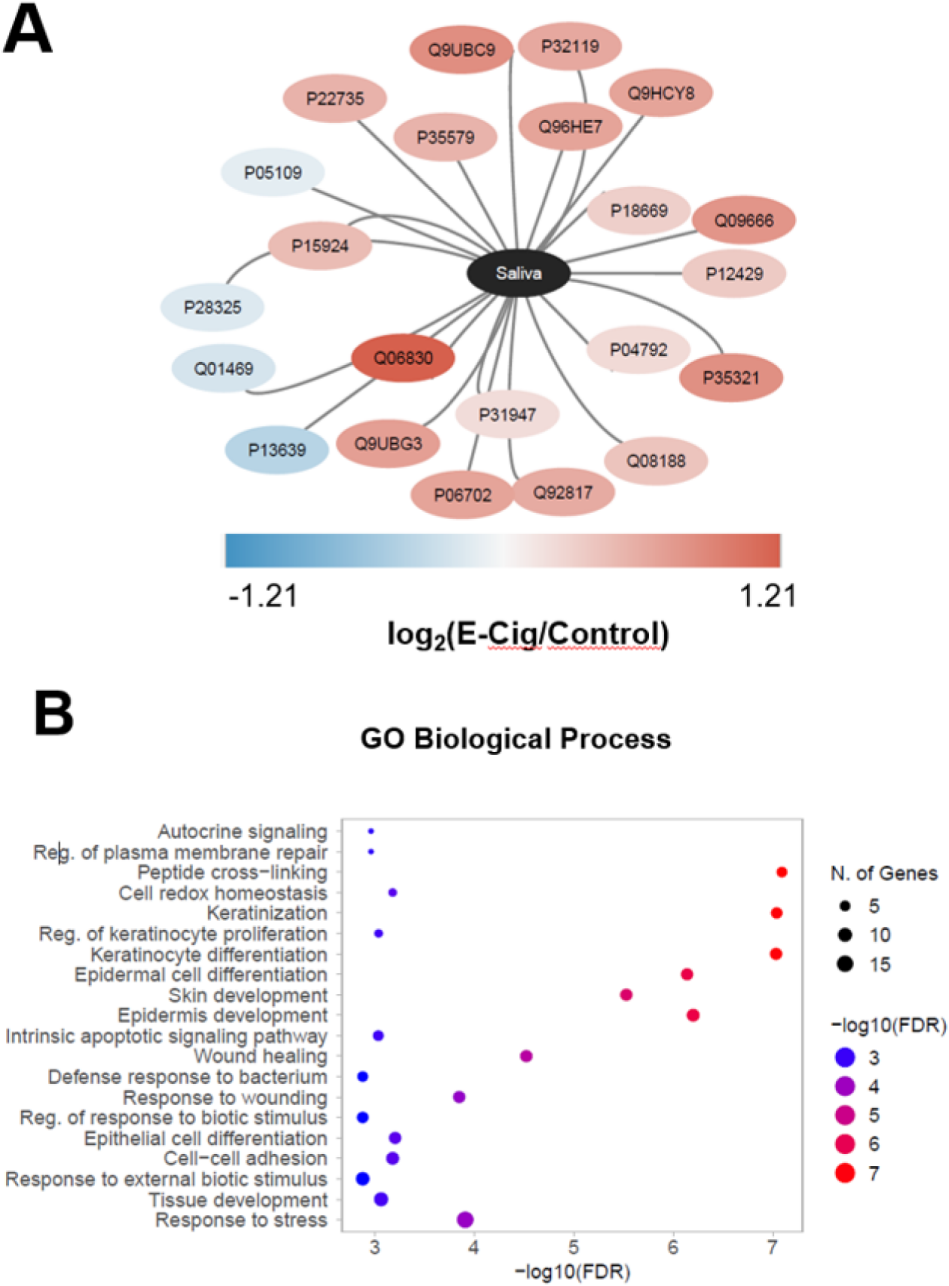
Analysis of salivary proteins. (A) Network representation of log_2_(fold change) values from the 22 statistically significant proteins (Wilcoxon–Mann–Whitney test). (B) GO enrichment analysis (biological process) for the 22 statistically significant proteins.

The functional enrichment analysis of the 22 significant proteins (Figure 2B) demonstrated a strong impact on pathways related to epithelial integrity and cellular response. The pathways with the highest statistical significance were Keratinization and Keratinocyte Differentiation, followed by Epidermal Cell Differentiation. Pathways such as Stress Response showed a high number of involved proteins, indicating that the protein changes are concentrated in mucosal defense and structuring processes.

The quantitative intensity results for the 22 differentially abundant proteins are detailed in Figure 3. For most proteins, expression in the EG group was observed to be significantly higher when compared to those in the CG (*p* < 0.05).

**Figure 3:**
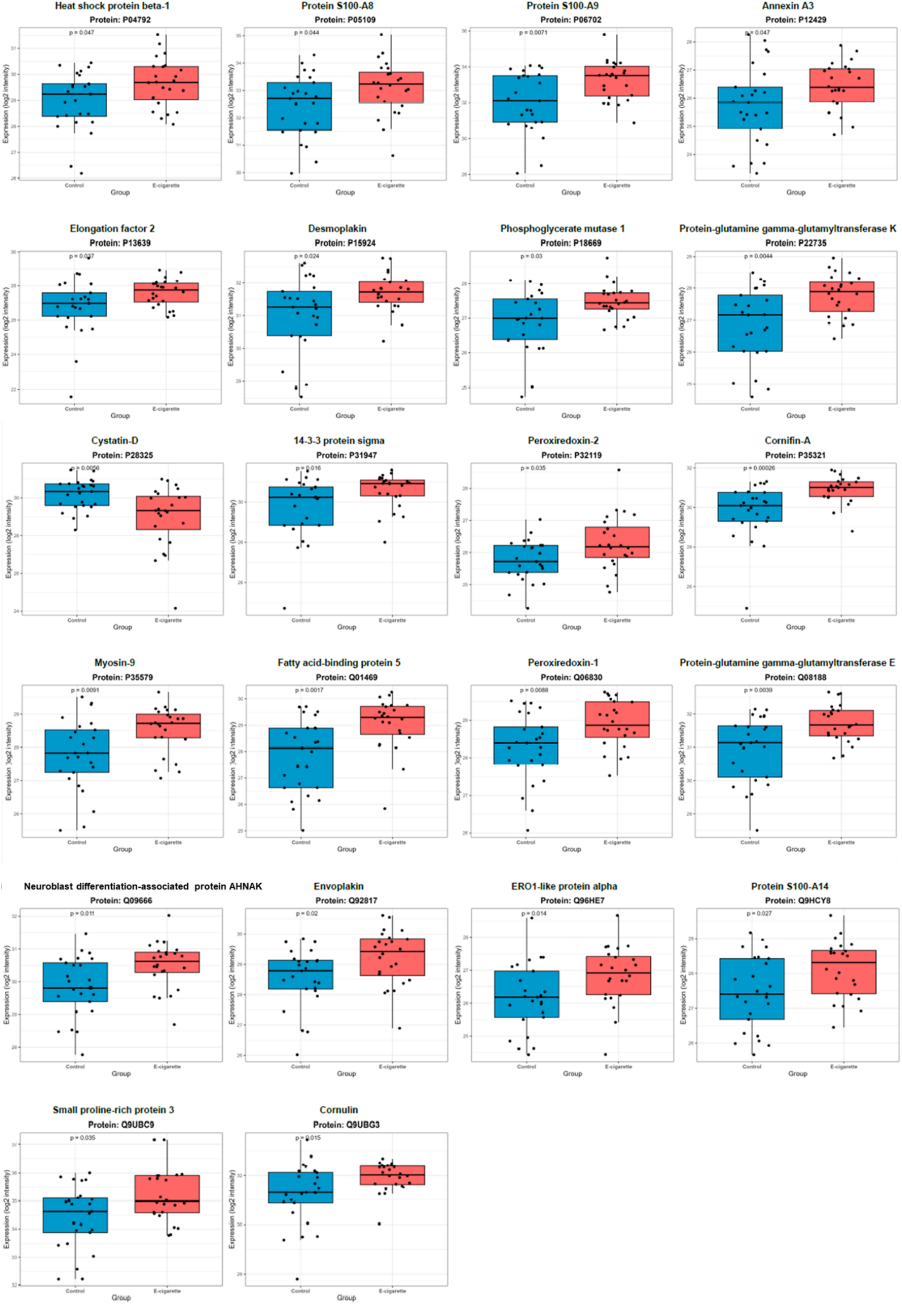
Proteins with statistically significant differences based on the *p*-value of the Wilcoxon–Mann–Whitney test (*n* = 22).

## Discussion

### Salivary Proteomics

Biomarkers are defined as measurable molecules that can be used as indicators of a normal or pathological biological process.^21^ Molecular markers can include DNA, RNA, metabolites, or proteins, and are useful in early diagnosis, prognosis, and staging of diseases.^22^ Biomarker analysis provides information on disease mechanisms and potentially affected tissues and systems, as well as indicating the individual susceptibility of patients.^23^

Previous studies have explored the salivary proteome of smokers, alcoholics, and users of substances such as crack.^24–26^ However, to date, no studies have specifically examined the specific salivary proteome of users of electronic cigarettes. Despite certain limitations, such as dietary variability among participants, our research team is among the first to explore this area. This study is particularly significant as it contributes to the identification of proteins and potential biomarkers associated with the early detection of harm linked to the use of electronic cigarettes.

The electronic cigarette is a device composed essentially of a lithium battery, which is responsible for heating the flavoring agents to high temperatures and generating the aerosol inhaled by users.^27^ This aerosol is made up of substances such as propylene glycol, formaldehyde, glycerin, variable concentrations of nicotine, and other compounds, many of which exhibit cytotoxic and carcinogenic properties either at room temperature or after heating.^28–30^

Studies indicate that components present in electronic cigarettes flavorings play a significant role in increasing the production of free oxidant radicals, posing a potential toxicological risk to users and impact the innate immune system, increasing the risk of infections.^31–33^

Given the need to understand short-term cellular damage and how the use of electronic cigarettes affects the abundance of proteins present in saliva, the salivary proteome emerges as an important field of study. Among the 22 statistically significant proteins identified in both groups, Peroxiredoxin-1 (PRDX1) was found to be up-regulated. Its primary function is to protect cells against oxidative stress caused by reactive oxygen species.^34^ Elevated levels of PRDX1 suggest increased oxidative stress in the oral environment, further challenging the misconception that electronic cigarettes are a safer alternative to conventional cigarette use.^35,36^ The strong representation of the stress-response pathway observed in the functional enrichment analysis is also supported by the increase in PRDX1.

Although the risk of oral and oropharyngeal cancer appears to be lower in electronic cigarette users compared with traditional smokers, the true risk factors associated with vaping have not yet been clearly established.^37^ Oxidative DNA lesions represent one of the stimuli capable of contributing to cancer development.^38^ Although the presence of PRDX1 is not a direct indicator of increased carcinoma risk in e-cigarette users—including oral squamous cell carcinoma—this finding highlights the harmful effects of e-cigarettes, which remain insufficiently studied in the long term.

Annexin A3 (ANXA3), which was also up-regulated in the study, emerges as a potential biomarker, showing differential expression across a wide range of tumors and being capable of sustaining proliferation, promoting invasion and metastasis, and inducing chemoresistance.^39^ However, although it demonstrates notable clinical value for risk stratification and early carcinoma detection, its clinical potential still requires further investigation, and no reports were found in the literature describing malignant lesions in the oral cavity associated with the presence of this protein.

Oxidative stress also acts as a potent signal for cellular defense and adaptation mechanisms.^40^ Proteins such as envoplakin and SPRR3, which are components of keratinocyte structure, are also up-regulated. The process of cellular differentiation and protection, which supports the increase of other pathways related to keratinization and keratinocyte differentiation, suggests a mucosal response to the environmental stress to which these tissues are exposed.^41^ This may represent a precursor of long-term alterations in oral health.

Altered expression of S100-A14 has been reported in multiple human malignancies, including oral squamous cell carcinoma, highlighting its relevance in this context.^42^ A study demonstrated that S100-A14 shows a gradual decrease in expression during the phenotypic transition from normal cells to dysplasia and carcinoma, and that its overexpression can inhibit the proliferation of oral squamous cell carcinoma-related cell lines compared with control cells.^43^

### Salivary Alterations

Experimental studies have shown that sweet-flavored products, in comparison with menthol or conventional tobacco flavors, tend to be more appealing and lead to higher levels of exposure, which may contribute to increased frequency and continued use.^44,45^ Elevated salivary cotinine concentrations indicate substantial nicotine exposure, which not only contributes to dependence but also enhances biofilm formation and microorganism viability.^46^ Therefore, the presence of high cotinine concentrations detected in the saliva of the participants in this study raises serious concerns regarding the potential long-term effects of electronic cigarettes on oral health.

Studies on the impact of electronic cigarette use on the characteristics of users’ saliva are still scarce. Although no differences were identified between the groups regarding sialometry, baseline pH, or buffering capacity, changes in saliva viscosity were observed. Salivary viscosity reflects the mucin content in saliva, an important factor for the protection and hydration of the oral mucosa.^47^ In the present study, the e-cigarette group exhibited lower viscosity compared with the control group, which may indicate changes in the composition of the samples. To our knowledge, no previous studies have specifically investigated this aspect.

Analysis of sialometry revealed that the e-cigarette group exhibited lower salivary flow values compared with the control group. This reduction may be linked to compounds commonly found in flavoring solutions, such as propylene glycol and glycerin, which are known to irritate the upper respiratory tract and promote drying of the mucous membranes.^48^

Previous studies have reported that conventional cigarette use can reduce salivary pH, creating a more acidic oral environment that favors dental demineralization.^49,50^ In the present study, no statistically significant differences were observed in salivary pH between groups; however, the mean pH among e-cigarette users was slightly higher than that of nonsmokers, underscoring the need for further research to better understand these effects.

Despite the increasing number of research studies that highlight the risks associated with the use of electronic cigarettes, significant knowledge gaps persist. The need for more studies and long-term monitoring of users is evident. However, the urgency of implementing preventive and educational measures alongside the widespread dissemination of accurate information remains crucial.

## Conclusions

The results of this study demonstrate that electronic cigarette use triggers measurable biological alterations in the oral environment. Changes in the salivary proteome, marked by the up-regulation of proteins involved in oxidative stress and epithelial differentiation, suggest early molecular responses to aerosol exposure.

Alterations in salivary characteristics, including high cotinine levels and reduced viscosity, further indicate disruptions that may compromise mucosal protection and overall oral homeostasis. Together, these results reinforce concerns about the long-term effects of vaping, which remain insufficiently understood. In parallel, the dissemination of accurate information and preventive guidance remains essential to counter the perception of electronic cigarettes as a harmless alternative.

## Materials and Methods

### Ethical Aspects

The present study was approved by the Human Research Ethics Committee (CEPH) of the Institute of Science and Technology of São José dos Campos (ICT-UNESP) (CAAE: 36911420.0.0000.0077, approval number: 4.397.780).

### Selection of Participants

The convenience sample was defined according to pre-established parameters.^20^ According to the Bonferroni correction, for a significance level of 0.01 and a power of 90%, the total sample size should be 48 patients. Accordingly, the 50 participants in the study were allocated into two groups:

1. Electronic Cigarette Group (EG): Composed of 25 regular and exclusive users of e-cigarettes for at least six months, without visible clinical changes in the oral mucosa.
2. Control Group (CG): Comprising 25 nonsmokers and nonusers of electronic cigarettes without visible clinical changes in the oral mucosa.

Study participants were enrolled between January and August 2022 from the cities of São José dos Campos, Mogi das Cruzes, and Jandira. Inclusion criteria required individuals to be at least 18 years old, to provide written informed consent, and to have no history of chronic systemic illnesses. For the e-cigarette group, eligibility additionally required a history of smoking cessation of at least two years. Exclusion criteria included dual use of conventional and electronic cigarettes, ongoing treatment for autoimmune conditions, a history of chronic systemic diseases or long-term medication use, pregnancy or breastfeeding, and any previous surgical, chemotherapeutic, or radiotherapeutic interventions.

To ensure the absence of lesions or infections, all participants underwent extraoral and intraoral examinations. Clinical parameters for all participants were assessed by measuring heart rate, oximetry, capillary blood glucose levels, and exhaled carbon monoxide (CO) concentration using the piCO+ Smokerlyzer device (Bedfont Scientific Ltd., United Kingdom).

### Sample Collection

Participants were instructed not to brush their teeth or consume food for 2 h and to abstain from drinking alcohol for 12 h prior to sample collection. To reduce oral debris, participants rinsed their mouths with distilled water for 1 min before sample collection.^15^

Sample collection occurred during scheduled time frames, either 9:00–11:00 a.m. or post prandially (2:00–4:00 p.m.). Saliva collection was conducted with participants seated upright in a calm and well-ventilated environment. Unstimulated saliva was collected for 5 min through expectoration into a sterile disposable plastic tube. Samples were immediately placed on ice and transported to the laboratory. Each sample was divided into aliquots and stored at −80 °C until further proteomic analysis.

### Shotgun Proteomic Analysis

The saliva samples were initially thawed at room temperature, vortexed for 10 s, and then centrifuged at 14,000 rpm for 10 min at 4 °C to remove all debris, including insoluble material, cell debris, and food particles. The supernatant was transferred to a new microtube and cell lysis buffer (2% CHAPS, 150 mM NaCl and 50 mM HEPES, pH 7.5) was added.

Saliva protein concentrations were determined using 5 μL of saliva sample, 250 μL of Bradford reagent (Sigma, St. Louis, MO, USA) and bovine serum albumin (Sigma, St. Louis, MO, USA) as a standard on a 96-well plate. The absorbance was measured using a 595 nm spectrophotometer, and the data were exported to Microsoft Excel (Microsoft Inc., Redmond, USA). The protein concentrations of the samples were calculated in μg/μL based on the linear equation derived from the standard curve.

A 100 μg portion of saliva protein was mixed with guanidine hydrochloride (GuHCl) prepared in 50 mM HEPES (pH 7.5) to achieve a final concentration of 3 M. Then 1,4-dithiothreitol (DTT) was added in 50 mM HEPES (pH 7.5) was added to a final concentration of 5 mM, and the mixture was incubated for 1 h at 65 °C to reduce disulfide bridges in proteins.

For the alkylation of free sulfhydryls, 2-iodoacetamide (IAA) was added in 50 mM HEPES (pH 7.5) to a final concentration of 15 mM. The samples were incubated for 30 min at room temperature in the dark. After this incubation, dithiothreitol (DTT) was added to a final concentration of 15 mM, and the samples were incubated for an additional 20 min at room temperature to quench excess IAA.

To remove salts from the protein denaturation step, protein precipitation was performed by adding 8 volumes of acetone at −20 °C and 1 volume of methanol at −20 °C. The samples were incubated at −80 °C for 2 h. After incubation, the samples were centrifuged at 14,000*g* for 10 min at 4 °C and the supernatant was discarded. The pellet was washed with 1 volume of methanol and centrifuged again at 14,000*g* for 10 min at 4 °C. This washing step was repeated twice more.

After the final centrifugation, the supernatant was discarded, and the pellet was allowed to air-dry at room temperature. The proteins were solubilized in 10 μL of 100 mM NaOH. Subsequently, 80 μL of H_2_O and 10 μL of 500 mM HEPES pH 7.5 were added. Finally, trypsin (Mass Spec grade, Promega) was added at a 1:100 (*w*/*w*) enzyme-protein ratio and the mixture was incubated at 37 °C overnight.

After incubation, formic acid was added to a final concentration of 5% to acidify the samples and inactivate trypsin. Peptide samples were desalted using C18 membranes (stage tips) mounted on P1000 pipet tips. The tips were equilibrated by adding 500 μL of methanol, followed by 500 μL of Milli-Q water.

For column conditioning, 500 μL of 0.1% formic acid was added. Once the membranes were prepared, the samples were applied. Desalting was performed after two washes with 0.1% formic acid. To elute the peptides, the column was washed twice with 50% acetonitrile in 0.1% formic acid, followed by a final wash with 100% acetonitrile to ensure complete elution of the peptides. The desalted samples were dried using a SpeedVac. After drying, the peptides were quantified using bicinchoninic acid (BCA) and subjected to mass spectrometry analysis.

### Mass Spectrometry Analysis

Samples were analyzed on an Orbitrap Exploris 480 mass spectrometer (Thermo Fisher Scientific, Bremen, Germany) coupled to a nanoscale liquid chromatograph nLC Vanquish Neo (Thermo Fisher Scientific). The peptide extracts were eluted from the column using a gradient of 5–30% solvent B (90% acetonitrile, 0.1% formic acid) for 75 min, 30% to 40% solvent B for 7 min, and 40–99% solvent B for 8 min at a flow rate of 300 nL/min. The electrospray source was operated at 2.1 kV.

The peptide mixture was analyzed by acquiring spectra in full MS mode with a resolution of 60,000 for the determination of MS1. The maximum injection time in automatic mode ranged from 400 to 1000 *m*/*z*. The 20 most intense peaks were automatically selected by Data-Dependent Acquisition (DDA) for subsequent acquisition of MS/MS spectra using two compensation voltages configured in the FAIMS system: −45 V and −60 V. MS/MS spectra were acquired with a resolution of 30,000, a maximum injection time of 50 ms. A dynamic exclusion of 30 s was applied.

### Bioinformatic Analysis

The raw data obtained from the shotgun mass spectrometer (RAW files) were processed using MaxQuant software (version 2.2.0.0). A false discovery rate (FDR) of 1% was required for protein and peptide-to-spectrum match identifications. Data were searched against a target database restricted to the taxonomy “*Homo sapiens* *”* (UniProt/SwissProt; 20,431 entries) combined with the sequences of 245 common contaminants and concatenated with the reverse versions of all sequences. Enzyme specificity was set to trypsin, with up to two missed cleavages allowed; cysteine carbamidomethylation was selected as a fixed modification, while methionine oxidation, glutamine/asparagine deamidation, and protein N-terminal acetylation were selected as variable modifications. The identification of the peptide was based on an initial mass deviation of 7 ppm for the precursor ion and the fragment mass tolerance was set at 0.02 Da. Label-free quantitation was performed using the MaxLFQ algorithm, with the “match between runs” feature enabled in MaxQuant. As is typical with complex proteomes such as those of vertebrates, peptides can be shared between homologous proteins or splice variants, leading to the formation of “protein groups”. The first protein entry was selected as the representative for each protein group in the “proteinGroups.txt” file generated by MaxQuant.

Quantitative values corresponding to the identified proteins were transformed by taking the logarithm (base 2) and then subjected to quantile normalization using the “preprocessCore” library available on the R/Bioconductor platform. Comparative analysis between experimental conditions was conducted using the Limma library, also available on the R/Bioconductor platform. Proteins with adjusted *p*-values ≤ 0.05 and log_2_(fold change) > 1 or < −1 were considered differentially expressed.

The software used in the functional enrichment of genes associated with identified proteins was ShinyGO (Ge et al., 2020—Available—http://bioinformatics.sdstate.edu/go/). Standard parameters were used, considering the FDR cutoff value <0.05.

## Supplementary Material





## Data Availability

The mass spectrometry proteomics data have been deposited to the ProteomeXchange Consortium via the PRIDE partner repository^51^ with the data set identifier PXD066133 and token cxz2DnBQr1h9.

## Notes

Table S3: Identified proteins with log_2_-transformed intensity values (MaxQuant); Table S4: Quantile-normalized log_2_-transformed intensity values used for downstream analyses; Table S5: Statistical analysis of consistently quantified proteins, including Wilcoxon–Mann–Whitney *U*-test *p*-values and log_2_(fold change) values, with significant proteins indicated (p ≤ 0.05) (XLSX)
Figures S1–S2: Protein correlation matrix and boxplots comparing Electronic Cigarette and Control groups; Table S1: Demographic, clinical, and alcohol consumption data; Table S2: Electronic cigarette consumption data (PDF)

## Abbreviations

ENDS: Electronic Nicotine Delivery Systems
EG: Electronic Cigarette Group
CG: Control Group
LC-MS: Mass Spectrometer Coupled to Liquid Chromatograph
