# Supplementary material for: Salivary Proteomics Reveals Oxidative Markers in E‑Cigarette Users
Source: J Proteome Res. 2026 Mar 9;25(4):1846–54. doi: 10.1021/acs.jproteome.5c00347 (PMC13054854; doi:10.1021/acs.jproteome.5c00347)
Supplement: Supplementary file 2 [file pr5c00347_si_002.pdf]

Supporting Information
Salivary Proteomics Reveals Oxidative Markers in E-Cigarette Users

Natalia Faria[1], Bruna Fernandes do Carmo Carvalho[1], Monica Ghislaine Oliveria Alves[1], Ismael Lima[2], Leo Iwai[2], Murilo Salardani[3], André Zelanis[3], Janete Dias Almeida[1]*

[1]São Paulo State University, Institute of Science and Technology (ICT-UNESP), 12245-000, São José dos Campos, São Paulo, Brazil.

[2]Laboratory of Applied Toxinology (LETA), Center of Toxins, Immune-Response and Cell Signaling (CeTICS), Butantan Institute, 05503-900, São Paulo, Brazil.

[3]Functional Proteomics Laboratory, Institute of Science and Technology, Federal University of São Paulo (ICT-UNIFESP), 12231-280, São José dos Campos, São Paulo, Brazil.

Corresponding author's email: janete.almeida@unesp.br

Table of contents:

Supplementary Table S1. Demographic, clinical and alcohol consumption of study participants.

Supplementary Table S2. Electronic cigarette consumption of study participants.

Supplementary Table S3. Identified proteins (xlsx).

Supplementary Table S4. Quantile-normalized log2 quantitative values for all the samples (xlsx).

Supplementary Table S5. Statistical analysis of the 92 proteins shared by all subjects from the two groups (xlsx).

Supplementary Figure S1. Correlation matrix of proteins identified in at least one sample from each group.

Supplementary Figure S2. Boxplots illustrating the comparison between the two groups.

Supplementary Table S1. Demographic, clinical and alcohol consumption of study participants. Data correspond to the same cohort described in Carvalho et al. Int. J. Mol. Sci. 2024, 25(21), 11750. Reproduced under the Creative Commons Attribution (CC BY 4.0) license (https://creativecommons.org/licenses/by/4.0/).

Variables	EG	CG	p-value
Participants	25 (100%)	25 (100%)	
Female	11 (44%)	11 (44%)	NA
Male	14 (56%)	14 (56%)	
Age (in years)	27 ± 7.57	26.80 ± 6.67	0.996
Self-reported race/skin color			
White	22 (88%)	20 (80%)	
Brown	3 (12%)	4 (16%)	NA
Black		1 (4%)	
Schooling			
High School	2 (8%)		
Ongoing Undergraduate	10 (40%)	13 (52%)	
Undergraduate	9 (36%)	1 (4%)	
Graduate	1 (4%)	11 (44%)	
UD	3 (12%)		
Phisical examination			
Heart rate	77.72 ± 14.26	81.88 ± 14.53	0.295
Capillary blood glucose (mg/dL)	96.21 ± 17.88	101.90 ± 13.19	0.121
Oximetry (% O_2)	96.76 ± 1.23	97.56 ± 1.04	0.042*
CO concentration (ppm)	2.12 ± 1.59	1.48 ± 0.92	0.015*
Saliva parameters			
Sialometry (mL/min)	0.90 ± 0.27	1.24 ± 1.69	0.782
pH	7.12 ± 0.67	7.04 ± 0.57	0.755
Viscosity (cm)	2.04 ± 1.36	2.68 ± 0.9	0.048*
Cotinine concentration (ng/mL)	46.88 ± 20.01	0.01 ± 0.04	<0.001*
Alcohol consumption			
AUDIT	7.76 ± 4.81	4.04 ± 2.34	0.003*
Low risk consumption	11 (44%)	23 (92%)	
Harmful consumption	8 (32%)	2 (8%)	NA
Moderate-severe disorder	2 (8%)		
UD	4 (16%)		
Average doses consumption			
1 to 2 doses	0 (0%)	10 (40%)	
3 to 4 doses	10 (40%)	6 (24%)	
5 to 6 doses	5 (20%)	4 (16%)	NA
7 to 9 doses	3 (12%)	3 (12%)	
≥ 10 doses	3 (12%)	0 (0%)	
UD	4 (16%)	2 (8%)	
Alcoholic beverages			
Beer	10 (40%)	13 (52%)	
Distilled drinks	10 (40%)	5 (20%)	NA
Wine	1 (4%)	5 (20%)	
UD	4 (16%)	2 (8%)	

Supplementary Table S2. Electronic cigarette consumption of study participants. Data correspond to the same cohort described in Carvalho et al. Int. J. Mol. Sci. 2024, 25(21), 11750. Reproduced under the Creative Commons Attribution (CC BY 4.0) license (https://creativecommons.org/licenses/by/4.0/).

Variables	EG
Participants	25 (100%)
E-cig use (in years)	2,13 ± 1,23
Former smoker (industrialized cigarettes)	6 (24%)
Abstinence of industrualized cigarettes (in years)	2,67 ± 0,82
E-cig consumption	
1 to 2 days a week	5 (20%)
3 to 4 days a week	7 (28%)
Daily	13 (52%)
E-cig frequency of use	
3 to 4 times a day	4 (16%)
5 to 6 times a day	6 (24%)
7 to 10 times a day	3 (12%)
> 10 times a day	12 (48%)
Vaporization time	
about 1 minute	10 (40%)
1 to 2 minutes	11 (44%)
3 to 5 minutes	4 (16%)
Flavorings	
Flavoring (in ml) per day	8,62 ± 12,65
Nicotine (in mg) per day	37,20 ± 59,95
Nicotine (in mg) per day - UD	5 (20%)
Flavoring types	
Fruits and sweet	12 (48%)
Fruits and mint	4 (16%)
Mint and Ice	5 (20%)
UD	4 (16%)
Simultaneously e-cig and alcohol use	
Yes	19 (76%)
Sometimes	2 (8%)
No	0 (0%)
UD	4 (16%)
Does drinking alcohol increase e-cig use?	
Yes	13 (52%)
Sometimes	4 (16%)
No	4 (16%)
UD	4 (16%)
How much alcohol use increases e-cig use?	
1 to 2 times more	6 (24%)
3 to 4 times more	8 (32%)
5 to 6 times more	2 (8%)
I can't say	5 (20%)
UD	4 (16%)

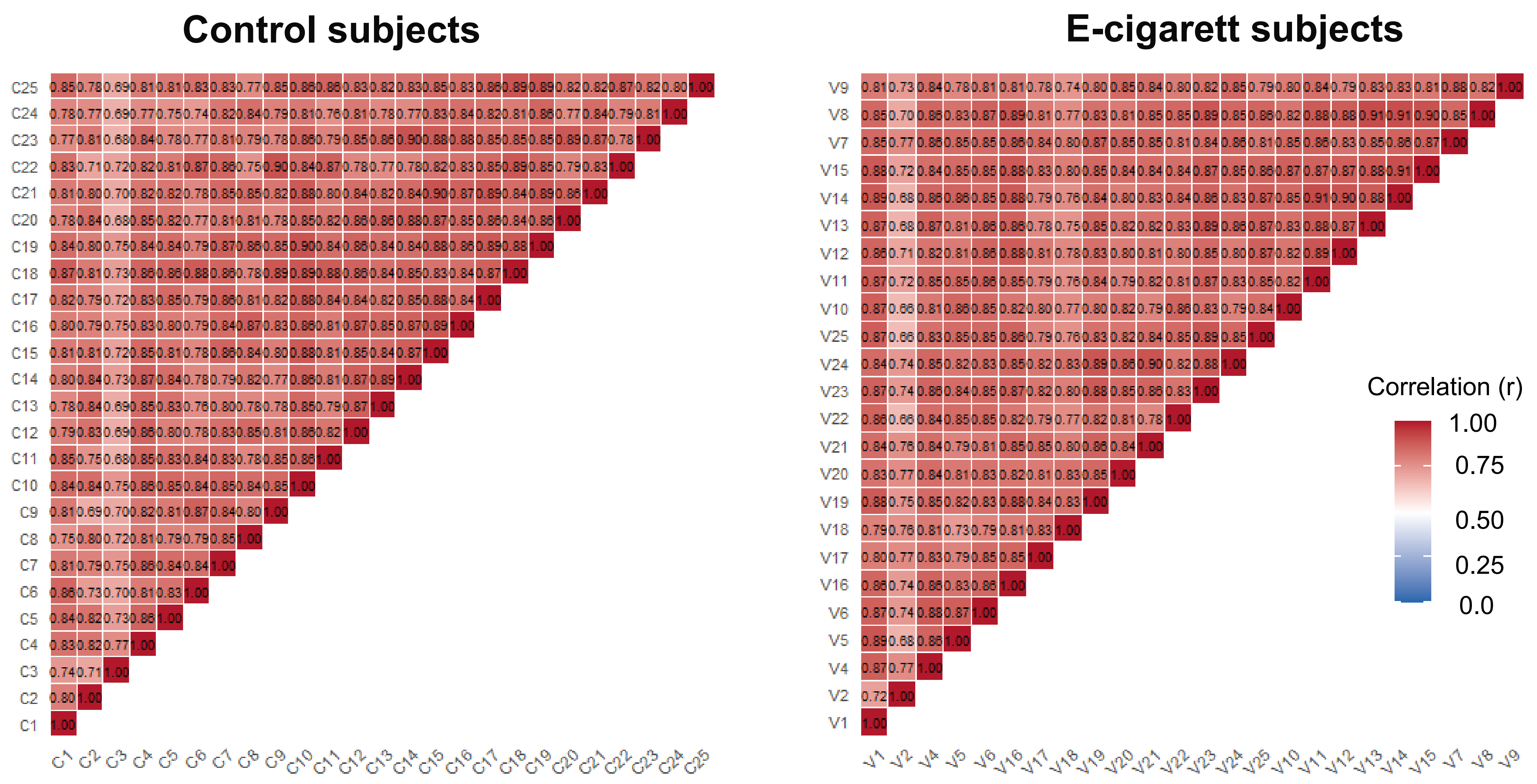

Supplementary Figure S1

The correlation matrix was constructed based on the normalized intensities of proteins identified in at least one sample from each group. The Pearson correlation coefficient was applied to measure the linear relationship between the protein abundance profiles of the samples, using the parameter use = "pairwise.complete.obs" to ignore missing values. The visualization was generated with ggplot2, representing the upper triangular matrix, with correlations color-coded and the numerical value overlaid on each cell.

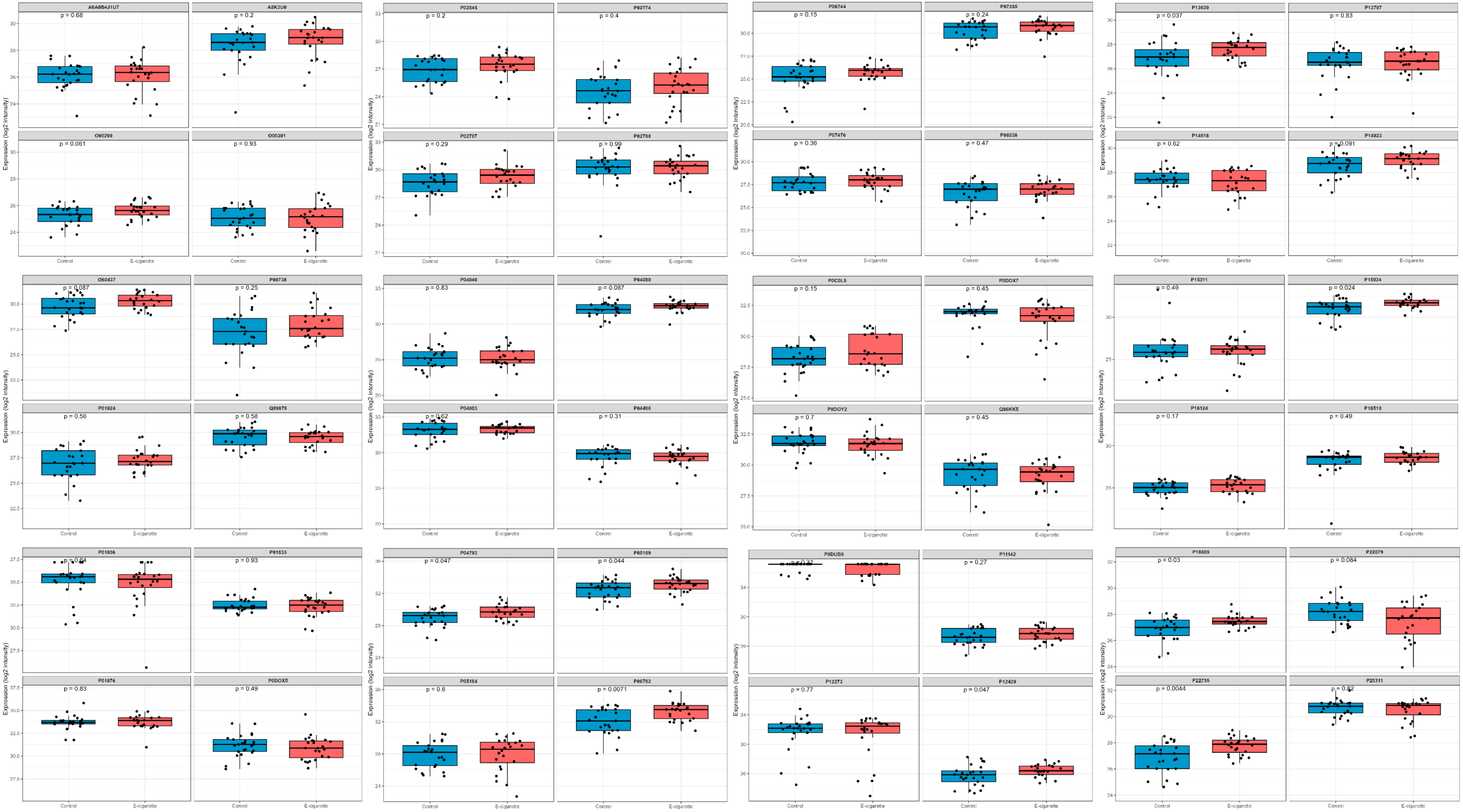
Expression (log2 intensity)
A0A0B4J1U7
p = 0.68
A8K2U0
p = 0.2
O00299
p = 0.061
O00391
p = 0.93
P02545
p = 0.2
P02774
p = 0.4
P02787
p = 0.29
P02788
p = 0.99
P06744
p = 0.15
P07355
p = 0.24
P07476
p = 0.36
P08238
p = 0.47
P13639
p = 0.037
P13797
p = 0.83
P14618
p = 0.62
P14923
p = 0.091
O60437
p = 0.087
P00738
p = 0.25
P01024
p = 0.56
Q99879
p = 0.58
P04040
p = 0.83
P04080
p = 0.087
P04083
p = 0.62
P04406
p = 0.31
P0C0L5
p = 0.15
P0DOX7
p = 0.45
P0DOY2
p = 0.7
Q96KK5
p = 0.45
P15311
p = 0.49
P15924
p = 0.024
P18124
p = 0.17
P18510
p = 0.49
P01036
p = 0.64
P01833
p = 0.93
P01876
p = 0.83
P0DOX5
p = 0.49
P04792
p = 0.047
P05109
p = 0.044
P05164
p = 0.6
P06702
p = 0.0071
P0DUB6
p = 0.31
P11142
p = 0.27
P12273
p = 0.77
P12429
p = 0.047
P18669
p = 0.03
P22079
p = 0.084
P22735
p = 0.0044
P25311
p = 0.82
Control
E-cigarette

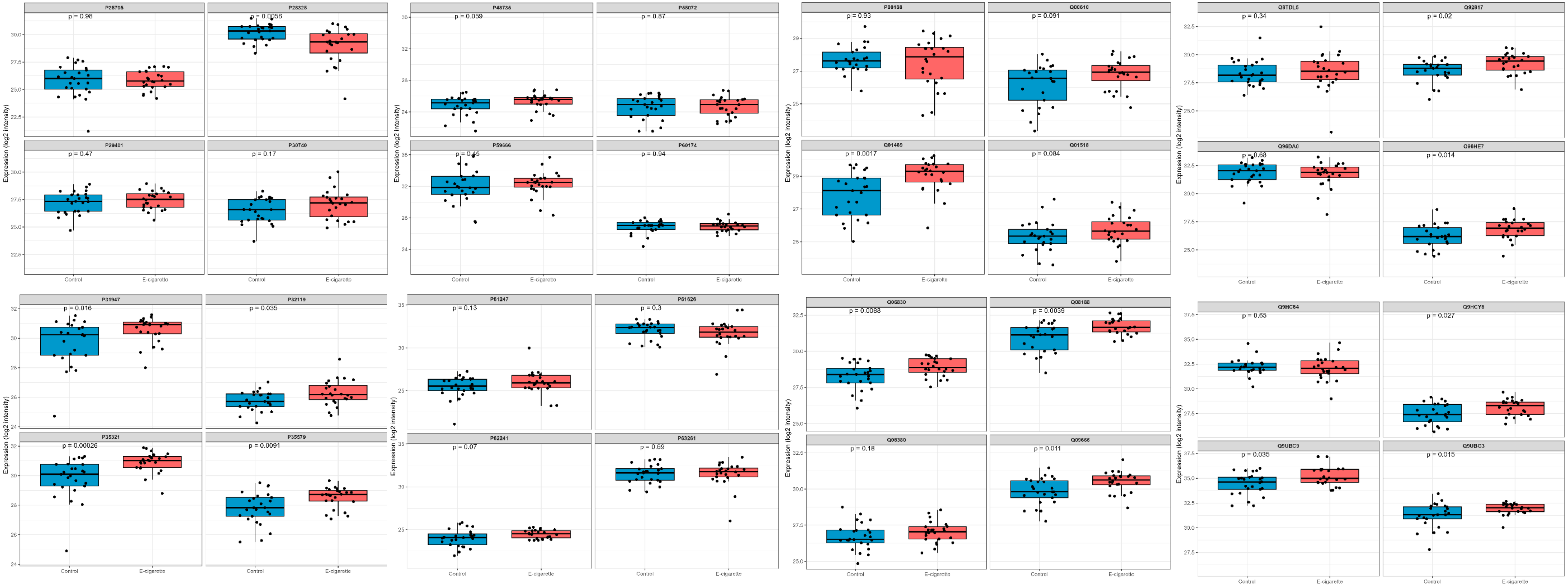

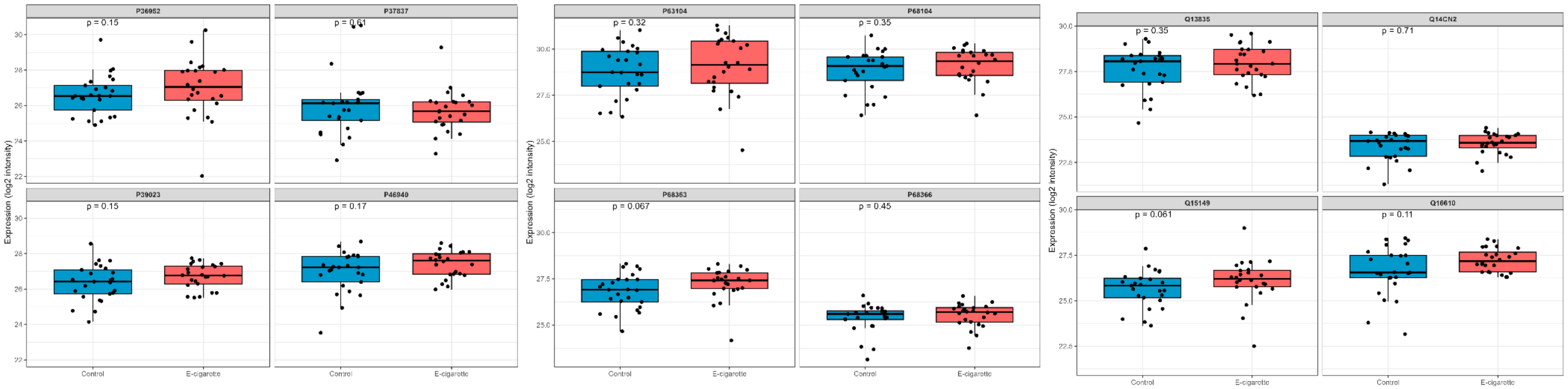

Supplementary Figure S2
Boxplots displaying the comparison of both groups (Control- blue and E-cig., -red) together with their associated p-values (Wilcoxon–Mann–Whitney test)
